# Early structural remodeling and deuterium oxide-derived protein metabolic responses to eccentric and concentric loading in human skeletal muscle

**DOI:** 10.14814/phy2.12593

**Published:** 2015-11-12

**Authors:** Martino V Franchi, Daniel J Wilkinson, Jonathan I Quinlan, William K Mitchell, Jonathan N Lund, John P Williams, Neil D Reeves, Kenneth Smith, Philip J Atherton, Marco V Narici

**Affiliations:** 1 MRC-ARUK Centre of Excellence for Musculoskeletal Ageing Research, School of Medicine, University of NottinghamDerby, UK; 2 School of Healthcare Science, Faculty of Science & Engineering, Manchester Metropolitan UniversityManchester, UK

**Keywords:** Exercise, hypertrophy, metabolism, muscle architecture, protein synthesis

## Abstract

We recently reported that the greatest distinguishing feature between eccentric (ECC) and concentric (CON) muscle loading lays in architectural adaptations: ECC favors increases in fascicle length (Lf), associated with distal vastus lateralis muscle (VL) hypertrophy, and CON increases in pennation angle (PA). Here, we explored the interactions between structural and morphological remodeling, assessed by ultrasound and dual x-ray absorptiometry (DXA), and long-term muscle protein synthesis (MPS), evaluated by deuterium oxide (D_2_O) tracing technique. Ten young males (23 ± 4 years) performed unilateral resistance exercise training (RET) three times/week for 4 weeks; thus, one-leg trained concentrically while the contralateral performed ECC exercise only at 80% of either CON or ECC one repetition maximum (1RM). Subjects consumed an initial bolus of D_2_O (150 mL), while a 25-mL dose was thereafter provided every 8 days. Muscle biopsies from VL midbelly (MID) and distal myotendinous junction (MTJ) were collected at 0 and 4-weeks. MPS was then quantified via GC–pyrolysis–IRMS over the 4-week training period. Expectedly, ECC and CON RET resulted in similar increases in VL muscle thickness (MT) (7.5% vs. 8.4%, respectively) and thigh lean mass (DXA) (2.3% vs. 3%, respectively), albeit through distinct remodeling: Lf increasing more after ECC (5%) versus CON (2%) and PA increasing after CON (7% vs. 3%). MPS did not differ between contractile modes or biopsy sites (MID-ECC: 1.42 vs. MID-CON: 1.4% day^−1^; MTJ-ECC: 1.38 vs. MTJ-CON: 1.39% day^−1^). Muscle thickness at MID site increased similarly following ECC and CON RET, reflecting a tendency for a contractile mode-independent correlation between MPS and MT (*P* = 0.07; *R*^2^ = 0.18). We conclude that, unlike MT, distinct structural remodeling responses to ECC or CON are not reflected in MPS; the molecular mechanisms of distinct protein deposition, and/or the role of protein breakdown in mediating these responses remain to be defined.

### Introduction

The most characteristic physiological adaptation to resistance exercise training (RET) is muscle hypertrophy. Typically, RET involves raising and lowering of weights via "concentric" (CON), shortening, and "eccentric" (ECC), lengthening, actions. Notably, ECC contractions yield greater force than CON (Katz 1939; Westing et al. 1991; Cook and McDonagh 1995), reaching values ~1.8-fold greater than the maximum isometric force in vitro and in situ (Katz 1939; Lombardi and Piazzesi 1990) and ~1.2 times in vivo (Westing et al. 1988; Aagaard et al. 2000). Also, ECC contractions are more efficient than CON, that is, the same absolute work is achieved at a lower metabolic cost (Abbott et al. 1952; Bigland-Ritchie and Woods 1976; Hoppeler 2014) and thus are considered particularly suitable for rehabilitation, exercising older frail individuals, and those with limited exercise capacity (LaStayo et al. 2000; Lindstedt et al. 2001).

The notion that ECC contractions produce greater force than CON contractions (thus implying the use of greater loads in RET program) has prompted some authors to suggest that ECC-type exercise could promote greater muscle hypertrophy and strength gains compared to CON (Roig et al. 2009). Nevertheless, the influence of contraction mode upon the degree of hypertrophy is contentious (Wernbom et al. 2007; LaStayo et al. 2014) with some reports of ECC being superior (Higbie et al. 1996; Hortobágyi et al. 1996, 2000; Seger et al. 1998; Vikne et al. 2006) and others showing similar hypertrophy (Jones and Rutherford 1987; Duncan et al. 1989; Blazevich et al. 2007; Nickols-Richardson et al. 2007; Moore et al. 2012; Franchi et al. 2014). Although our previous findings support the observations of similar muscle hypertrophy induced by CON and ECC when matched for relative maximal load (Reeves et al. 2009; Franchi et al. 2014), we highlighted that the key difference between ECC and CON adaptations is in the specific architectural remodeling. Specifically, ECC training promoted increases in fascicle length (Lf), whereas CON preferentially increased pennation angle (PA) (M. V. Narici, M. V. Franchi and C. N. Maganaris, submitted). These findings suggest contraction-specific hypertrophy: longitudinal muscle growth (i.e., Lf increase) primarily with ECC (Seynnes et al. 2007; Potier et al. 2009) due to the addition of new sarcomeres in series (Williams and Goldspink 1971; Williams 1990; Lynn and Morgan 1994), and transversal hypertrophy highly in CON, due to increases in PA reflecting the addition of sarcomeres in parallel (Gans 1982; Kawakami et al. 1993; Narici and Maganaris 2007). Furthermore, disparities in the regional distribution of changes in anatomical cross-sectional area (ACSA) were also found (Franchi et al. 2014). While ECC resulted in preferential hypertrophy in distal region of m. *vastus lateralis* (VL), CON did so in the midbelly. Therefore, distinct architectural and spatial adaptations dominate distinct physiological responses to CON versus ECC contractions.

While it is known that muscle hypertrophy is the result of (cumulative) postexercise increases in muscle protein synthesis (MPS) (Atherton and Smith 2012), the response of MPS to CON versus ECC exercises has been subject of study of only a few investigations (Phillips et al. 1997; Cuthbertson et al. 2006). In these studies, similar increases in MPS were reported between CON and ECC (not matched for work) at 3, 24, and 48 h after exercise (Phillips et al. 1997). Similarly, MPS was not different following 12-min step-up (CON) or step-down (ECC) exercise (not work matched). However, others (Moore et al. 2005) reported that, when ECC versus CON exercise was matched for work (maximally on an isokinetic dynamometer), increases in MPS were greater 4.5 h after exercise following ECC compared to CON exercise, albeit with no differences after 8.5 h. As such, the metabolic responses to CON and ECC in the context of hypertrophy and distinct architectural adaptations remain poorly defined.

By using recently developed $D_2O$ tracers approaches (Previs et al. 2004; Gasier et al. 2010; Robinson et al. 2011; Wilkinson et al. 2014), the aim of the present study was to investigate whether the different patterns of skeletal muscle remodeling with ECC versus CON training were associated with differences in MPS, and through a novel biopsy approach at midbelly and in proximity to the myotendinous junction of the vastus lateralis (VL) muscle, if the previously reported heterogeneity in muscle hypertrophy along the muscle was reflected by regional differences in MPS. We hypothesized that MPS would preferentially increase at midbelly in response to CON RE, and at distal VL in response to ECC RE.

## Methods

### Ethics and volunteer characteristics

We recruited 10 recreationally active (this being defined as not currently, or recently undertaking in formal exercise programs) healthy young men (aged 23 ± 4 years; body mass, 83 ± 4 kg; height 184 ± 2 cm and body mass index [BMI] 24 ± 1 kg m$^2$). Volunteers were clinically screened by means of a medical questionnaire, to exclude sufferers of joint disease and metabolic, respiratory, or cardiovascular impairments. All subjects provided written informed consent. This study was approved by The University of Nottingham Ethics Committee and was compliant with the Declaration of Helsinki.

### Exercise training protocol

RET was carried out with a customized leg-press machine (Technogym, Gambettola Italy) specifically adapted to perform exclusively either ECC or CON contractions (refer to Franchi et al. 2014). Participants completed 4 weeks RET (load was set at 80% of CON or 80% ECC 1RM) with volunteers training both legs: each leg was randomly assigned to a specific contractile mode so one leg performed CON only and the contralateral only ECC. As in our previous study (Franchi et al. 2014), matching the training load for the same relative intensity (80% 1RM ECC and 80% 1RM CON) required similar levels of neural drive, based on integrated EMG activity of the VL, that is, CON and ECC were performed at the same level of neural activation, a fundamental of the force–velocity relationship (Bigland and Lippold 1954; Chow and Darling 1999). The protocol for RET was four sets of 8–10 repetitions with 1 min rest between sets with duration of contractions of ~2 sec CON versus ~3 sec ECC, as reported previously (Franchi et al. 2014).

### Muscle architecture

Muscle architecture was evaluated by analysis of ultrasonographic images (US) of VL muscle obtained at rest using B-mode ultrasonography (Mylab 70, Esaote Biomedica, Italy), with a 100 mm, 10–15 MHz, linear array probe. Resting US images were taken while the participant was lying supine on a bed for examination (corresponding to full knee extension). The US images were taken at the middle of VL length, with the midpoint of the probe placed longitudinally exactly at 50% of VL length and on the midsagittal line of the muscle. The transducer was then aligned in the fascicle plane to capture an optimal portion of fascicles (Reeves et al. 2009). Images were collected and digitally analyzed by the same unblinded operator. Quantification of muscle architectural adaptations, as assessment of fascicle length (Lf), pennation angle (PA), measured as the intersection between fascicles and the deep tendon aponeurosis values, and muscle thickness (MT), measured as the perpendicular distance between the superficial and the deep tendon aponeurosis, were performed by using ImageJ 1.42q software (National Institutes of Health, Bethesda, MD). The visible portion of the fascicle length was directly assessed and when fascicles extended off the ultrasound window, an estimation of the nonvisible part was then performed using a linear extrapolation of fibers and aponeuroses (Erskine et al. 2009; Franchi et al. 2014). Muscle thickness (MT) was measured as it was previously established as an indicator of muscle mass (Miyatani et al. 2002; Takai et al. 2013; Abe et al. 2015) and thus a valid index of RET-induced hypertrophy.

*Vastus lateralis* architecture was assessed on both legs using US at the start of the training period and on the final day of the study (4 weeks after the first RET training session). One repetition maximum (1RM) was assessed for both ECC (1RM ECC) and CON (1RM CON) legs using the same protocol as described in our previous study (Franchi et al. 2014); 1RM was then rechecked on the first training day before the start of exercise.

### Muscle biopsies at the midbelly and proximal to the distal myotendinous junction

Biopsies were taken with the conchotome technique (Dietrichson et al. 1987) from the VL muscle of both ECC and CON exercise-trained thighs, and at two sites, a "DISTAL" site, close to the myotendinous junction (MTJ) and at a "MID" site, close to midbelly on day 0 (basal, pretraining) and again after 4 weeks, that is, in total eight biopsies were taken per participant. The MID site of VL was identified as that region corresponding to maximum VL cross-sectional area (CSA), that is, 50% of VL length. Basal biopsies were taken on the midsagittal line of the muscle in the midbelly (MID) and 15 mm anterior to the midsagittal line at 40 mm from the MTJ (DISTAL). Subsequent biopsies were performed at week 4, 30-mm posterior to the basal DISTAL biopsy and 30-mm proximal to the basal MID. This represents, to the best of our knowledge, the first human in vivo study to collect tissue from close to the MTJ. All biopsies were performed under local anesthesia (1% Lignocaine, B. Braun Melsungen, Germany) using an aseptic technique. Ultrasound scanning with Philips iU22 and 40 mm Philips L9-6 linear array transducer (Philips Healthcare, Reigate, UK) permitted determination of MTJ and muscle boundaries. Reduced muscle CSA in the DISTAL region necessitated US guidance throughout infiltration of local anesthesia and execution of conchotomy. The basal biopsies were collected prior the start of the exercise period, while the postexercise biopsies were collected in a window between 60 and 90 min after the last exercise bout or the training protocol.) Muscle was rapidly dissected free of fat and connective tissue, washed in ice-cold saline solution and frozen in liquid $N_2$, and stored at $-80°C$ until further analysis. A detailed schematic of the study protocol is provided in Figure 1.

### Deuterium in body water and deuterated alanine incorporation into myofibrillar protein

Immediately post biopsy on day 0, participants provided a saliva sample (collected in sterile plastic tubes) and ingested a single 150 mL oral bolus of $D_2O$ (70 Atoms%, Sigma-Aldrich, Poole, UK), labeling the body water pool to ~0.2%. To monitor body water enrichment throughout the study, each participant was required to provide a single daily saliva sample (for the first 8 days, then 2–3 times a week from day 9 until the end of the study, i.e., week 4) collected at midday at least 30 min after their last meal or drink. These were collected in sterile plastic tubes and kept refrigerated, participants were asked to bring these

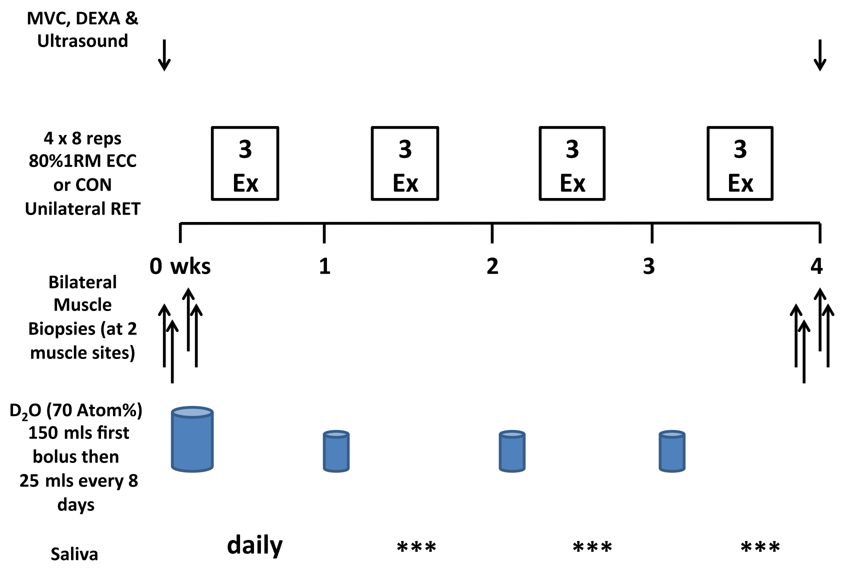

**Figure 1.** Study design scheme.

to the following training session. Upon receipt of saliva samples, they were immediately cold centrifuged at 16,000 *g* to remove any debris that may be present; they were then aliquoted into 2 mL glass vials and frozen at −20°C until analysis (as described by Wilkinson et al. 2014). Body water enrichment was topped up weekly with the ingestion of 25 mL of $D_2O$ (70% AP).

Body water enrichment was assessed by direct liquid injection of the saliva samples (0.1 $\mu$L volume) into a High Temperature Conversion Elemental Analyser (TC/EA; Thermo Finnigan, Thermo Scientific, Hemel Hempstead, UK) connected to an Isotope Ratio Mass Spectrometer (IRMS, Delta V Advantage, Thermo, UK). To reduce the effect of carryover between different samples, each saliva sample was injected for four times. A validation of the accuracy of the TC/EA for measuring body water enrichment from saliva has been reported in a previous publication from our laboratory (Wilkinson et al. 2014). The myofibrillar fraction was isolated from ~30 to 40 mg of muscle using our standard approach (Wilkinson et al. 2014). Briefly, the tissue was homogenized in ice-cold homogenization buffer (50 mmol/L Tris-HCl [pH 7.4], 50 mmol/L NaF, 10 mmol/L $\beta$-glycerophosphate disodium salt, 1 mmol/L EDTA, 1 mmol/L EGTA, 1 mmol/L activated $Na_3VO_4$; all from Sigma-Aldrich, Poole, UK) and a complete protease inhibitor cocktail tablet (Roche, West Sussex, UK) at 10 $\mu$L $\mu g^{-1}$ of tissue. Homogenates were rotated for 10 min and the supernatant collected by centrifugation at 13,000 *g* for 5 min at 4°C. The myofibrillar pellet was solubilized in 0.3 mol/L NaOH and separated from the insoluble collagen by centrifugation and the myofibrillar protein was precipitated with 1 mol/L perchloric acid (PCA). Protein-bound AAs from myofibrillar were released using acid hydrolysis by incubating in 0.1 mol/L HCl in Dowex $H^+$ resin slurry overnight before being eluted from the resin with 2 mol/L $NH_4OH$ and evaporated to dryness. The AAs were then derivatized as their *n*-methoxycarbonyl methyl esters (MCME) according to the protocol of Husek and Liebich (1994), with slight modification. The dried samples were resuspended in 60 $\mu$L of distilled water and 32 $\mu$L of methanol, following brief vortex, 10 $\mu$L of pyridine and 8 $\mu$L of methylchloroformate were added. Samples were vortexed for 30 sec and left to react at room temperature for 5 min. Newly formed MCME AAs were extracted into 100 $\mu$L of chloroform, remaining water was removed from the sample with addition of a molecular sieve. Incorporation of deuterium into protein-bound alanine was determined by gas chromatography–pyrolysis–isotope ratio mass spectrometry (Delta V Advantage, Thermo Scientific, Hemel Hempstead, UK).

### Calculation of fractional synthetic rate (FSR)

The fractional synthetic rate (FSR) of myofibrillar (MyoPS) protein synthesis was determined from the incorporation of deuterium-labeled alanine into protein, using the enrichment of body water (corrected for the mean number of deuterium moieties incorporated per alanine, 3.7) as the surrogate precursor labeling between subsequent biopsies (i.e., between 0 and 4 weeks RET).

In brief, the standard equation is as follows:FSR (% $day^{-1}$) = $-\text{Ln}[-1[(APE_{Ala})/(APE_P)]/t]$, where $APE_{Ala}$ = deuterium enrichment of protein-bound alanine, $APE_P$ = precursor enrichment, and *t* represents the duration of time between biopsies, following the same rationale as described by Brook et al. (2015). Absolute synthesis rates (ASR) have been calculated (average between MID and MTJ muscle sites values) after 4 weeks of RET, following a similar design reported in Brook et al. (2015) manuscript. The equation used to calculate ASR was the following:ASR ($g*day^{-1}$) = (FSR/100)* TFFM* (12.4/100), where FSR is the myofibrillar fractional synthetic rate between 0 and 4 weeks, TFFM is the thigh fat-free mass calculated by DXA, and 12.4 represent the myofibrillar protein content estimation in TFFM as previously used by MacDonald et al. (2013).

#### Assessment of maximum voluntary contraction (MVC)

Maximum isometric knee extensor torque was assessed unilaterally via isokinetic dynamometry (Isocom, Isokinetic Technologies, Eurokinetics, UK). Volunteers performed isometric contractions at different knee joint angles: 90° to 50°, with full extension corresponding to 0°. Subjects were seated (hip angle: 85°; hip angle at supine position: 0°) secured into position using straps across the chest. The lower leg was strapped to the pad of the Isocom lever arm and the knee joint center of rotation was aligned with the dynamometer fulcrum. Contractions lasted 4 sec, with a rest period of 30 sec between contractions, and 90 sec between knee joint angle changes. Maximum isometric torque was assessed at two different time points (0 and 4 weeks).

#### Statistical analyses

All data are presented as mean ± SEM. Differences within groups (contraction mode × time) were detected by repeated measures with a two-way (contraction mode × time) factorial analysis of variance with a Bonferroni correction using GraphPad Prism software (Version 6, La Jolla, San Diego, CA). The delta (Δ) training values (% increases) were tested between groups using independent *t* tests. Linear regression analyses were assessed by Pearson's correlation coefficient. The alpha level of significance was set at $P < 0.05$.

### Results

#### Muscle function and training load

As expected in response to the RET protocol, the ECC training load was greater than CON throughout the study ($P < 0.0001$) (Fig. 2A). At the end of the RET period, the increment of training load was 21 ± 4% for the ECC leg ($P < 0.001$), whereas 18 ± 5% for the CON leg (P < 0.001). Changes in isometric MVC are shown in Figure 2B. Both ECC and CON RET limbs yielded identical increases ($P < 0.01$) in isometric MVC (11 ± 0.02% ECC vs. 11 ± 0.03% CON).

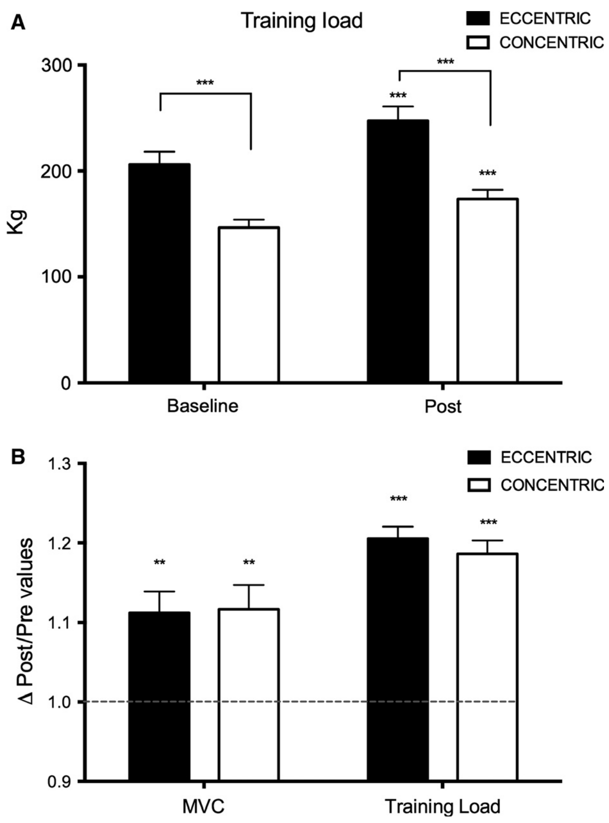

**Figure 2.** (A) Training load used during leg-press exercise, changes at baseline and after 4 weeks for ECC and CON loading paradigms; (B) Post/Pre value ratio of isometric MVC and the training load used after 4 weeks of either ECC or CON training protocol. $Y = 1$ represent the baseline value. $^{**}P < 0.01$, $^{***}P < 0.001$. Values are presented as means ± SEM.

#### Muscle morphology and architecture

Changes in muscle architecture (i.e., fascicle length, *Lf*, and pennation angle, *PA*) and thickness (MT) were investigated at baseline and after 4 weeks of unilateral ECC and CON RET regime. After 4 weeks RET, Lf significantly increased in the ECC trained leg (5 ± 0.6%, $P < 0.001$) and considerably more ($P < 0.01$) than in the CON RET leg (2 ± 0.6%, $P < 0.05$) (Fig. 3A). Conversely, CON RET exhibited greater increases in PA (7 ± 0.9%, $P < 0.001$, time effect) than the ECC RET leg (2.7 ± 0.5%) (Fig. 3A). Muscle thickness (MT) (a proxy of muscle cross-sectional area) (Fig. 3A) increased to a similar extent in the ECC and CON legs (7.5 ± 1.6% ECC vs. 8.4 ± 1.4% CON, $P < 0.001$).

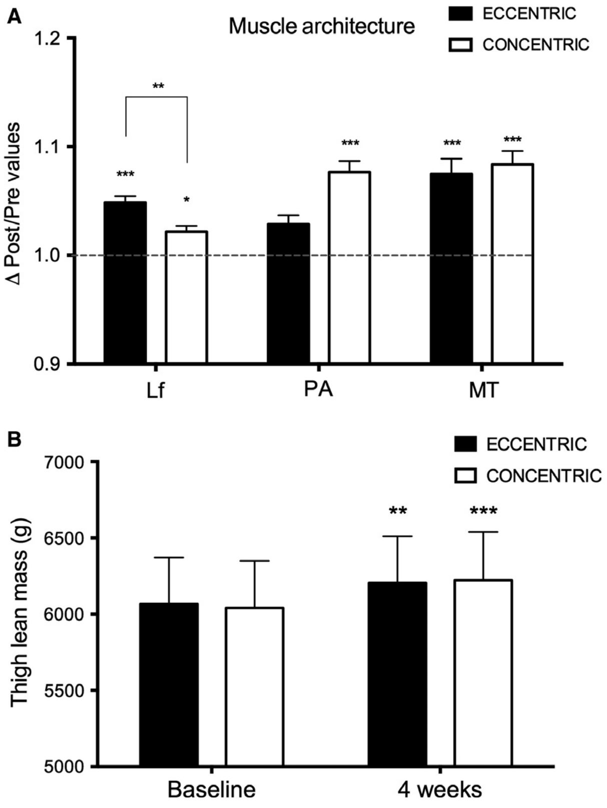

**Figure 3.** (A) Post/Pre value ratio of fascicle length, pennation angle, and muscle thickness after 4 weeks of either ECC or CON training protocol. $Y = 1$ represent the baseline value. *$P < 0.05$, **$P < 0.01$, ***$P < 0.001$. Values are presented as means ± SEM. (B) Thigh lean mass increases assed by DEXA after 4 weeks of either CON or ECC RET. **$P < 0.01$, ***$P < 0.001$. Values are presented as means ± SEM.

### Muscle mass (DXA)

Thigh lean mass (Fig. 3B) increased after both ECC and CON RET (2.3 ± 0.5% vs. 3 ± 0.6%; $P < 0.01$ and $P < 0.001$, respectively) with no group differences being evident.

### Muscle protein synthesis

Body water enrichment is presented in Figure 5A. After an initial priming bolus of 150 mL of 70% $D_2O$, body water was enriched up to 0.2 ± 0.01% and was maintained between 0.14 ± 0.005% and 0.05 ± 0.004% throughout the duration of the study. Body water followed an exponential decay, resulting in a decline of ~0.01Atom% $day^{-1}$ in the first 2 weeks, similar to data previously reported by our group (Wilkinson et al. 2014). In the last 2 weeks of the investigation, the body water enrichment decay resulted in ~0.0045Atom% $day^{-1}$

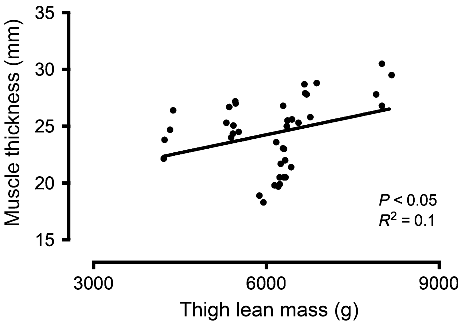

**Figure 4.** Linear regression analysis between muscle thickness and DEXA values after 4 weeks of either CON or ECC RET. $R^2 = 0.1$, $P < 0.05$.

reaching a pseudo steady state ranging 0.09 ± 0.007% and 0.05 ± 0.004%.

Figure 5B shows the daily myofibrillar fractional synthetic rates (FSR) for ECC and CON training, evaluated across 0–4 weeks of the RET program. After 4 weeks of RET, both ECC and CON trained limbs showed similar FSR values (MID-ECC: 1.42 ± 0.06% $day^{-1}$ vs. CON: 1.4 ± 0.05% $day^{-1}$; MTJ-ECC: 1.38 ± 0.05% $day^{-1}$ vs. CON: 1.39 ± 0.08% $day^{-1}$), with no statistically differences between sites and contractile mode.

The ASR values obtained for ECC and CON trained leg were 11.3 ± 0.8 and 11.1 ± 0.8 (g*$day^{-1}$), respectively, resulting in line with the one previously reported by Brook et al. (2015).

### Correlations

A significant positive linear relationship was found between MT and thigh lean mass ($R^2 = 0.1$, $P < 0.05$) (Fig. 4). Correlations between MPS (in terms of FSR $day^{-1}$) and muscle architecture features (MT, Lf, PA) were performed (Fig. 6). A trend for positive linear relationship was found between FSR and MT at 4 weeks (Fig. 6A), yet not statistically significant ($R^2 = 0.18$; $P = 0.07$). No significant correlations were found between MPS (FSR) and Lf or PA (Fig. 6B and C).

## Discussion

We investigated whether the morphological changes to ECC and CON training were related to long-term metabolic adaptations (i.e., MPS). To date, this is the first study that not only quantifies chronic MPS after ECC versus CON training regimes, but also attempts to identify metabolic explanations underlying distinct muscle architectural adaptations to ECC and CON resistive training. Furthermore, to the best of our knowledge, this is the first investigation into the effects on MPS in response to different loading paradigms collecting biopsies at two different sites (midbelly: MID site, and closer to the myotendinous junction: MTJ site) in the same muscle (VL). To compare the effects of ECC and CON, volunteers trained using an internally controlled bilateral approach, that is, one leg exclusively performing CON contractions, and the contralateral ECC. The unilateral – within-subject – design RET model, is well established and has been adopted by several previous studies either in the knee extensors (Kim et al. 2005; Moore et al. 2005; Cuthbertson et al. 2006; Kostek et al. 2007; Kumar et al. 2009) or in the elbow flexors (West et al. 2010; Moore et al. 2012). The advantages of such a design are the minimizing of variability in the training responses within groups (Hubal et al. 2005; Moore et al. 2012), and that the hypertrophic responses to RET are the result of localized muscular adaptations (West et al. 2010). Finally, our protocols were matched for relative load and neural drive (EMG), following a design pioneered by our research group (Reeves et al. 2009; Franchi et al. 2014). Such a tight control over the study design permits robust conclusions to be made.

Notably, ECC and CON RET produced similar increases in muscle thickness (Fig. 3A) and thigh lean mass measured by DXA (Fig. 3B). A positive correlation has been also found between MT and DXA (Fig. 4) (supporting even more the notion that MT is an indicator of muscular hypertrophic changes). Similar results in muscle CSA responses to ECC and CON of have been reported previously (Blazevich et al. 2007) after 10 weeks (matched for work using isokinetic contractions), and 12 weeks (Jones and Rutherford 1987) at 80% of the CON 1RM (leg extension machine). In the present study, although the ECC leg trained with higher loads compared to CON (but with matching EMG activity), this resulted in neither greater hypertrophy nor strength. These observations are supported by the findings of Moore et al. (2012), who showed similar increases in biceps CSA and MVC in response to 9 weeks of either CON or ECC isokinetic contractions, which were work matched. Altogether, the present findings and those previously cited (Blazevich et al. 2007; Moore et al. 2012) suggest that whether one adopts work-matched or neural drive-matched training regimes, a similar increase in muscle mass and strength is obtained with ECC and CON RET.

Although the changes in muscle thickness produced by the two loading regimes were very similar, changes in Lf and PA were contraction mode specific. Indeed as we previously reported, increases in Lf were greater following ECC than CON (5% vs. 2%); conversely, PA increased more following CON than ECC (8% vs. 3%). It is known that striated muscle fiber postnatal longitudinal growth is achieved by the addition of new sarcomeres in series and it is thought this occurs at the end of the muscle fibers (Goldspink 1968; Williams and Goldspink 1971). This also occurs in response to passive and/or intermittent stretch (Williams et al. 1988; Williams 1990) and lengthening regimes (Proske and Morgan 2001; Morgan and Proske 2004). Conversely, dynamic shortening contractions promote limited longitudinal growth or even loss of sarcomeres in series (uphill running: concentric contractions) in rats (Butterfield et al. 2005), instead favoring increased deposition of sarcomeres in parallel (i.e., increasing PA). Thus, chronic exposure to lengthening versus shortening reveals divergent remodeling patterns and, according to our data, these adaptations occur at an earlier stage of ECC and CON RET than previously defined.

These distinct architectural adaptations to CON as opposed to ECC training might suggest that the contraction-specific differential growth of skeletal muscle may be controlled by different responses of muscle anabolism (differential stimulation of new contractile material synthesis, i.e., MPS). On this basis, it is also legitimate to question whether these remodeling mechanisms could differ along the muscle length, as regional hypertrophy may be differently governed in a location-specific manner. Indeed, previous studies have investigated differences in MPS responses to lengthening and shortening contractions (Phillips et al. 1997; Moore et al. 2005; Cuthbertson et al. 2006). The main findings from these studies suggest MPS does not yield significant differences in response to the two mechanical stimuli, although ECC may result in different temporal stimulation of MPS versus CON. Nonetheless, a single bout of RET may not provide a "predictive" readout of ensuing muscle hypertrophy (Atherton and Smith 2012; Murton and Greenhaff 2013; Mitchell et al. 2014): the presence of acute-only MPS data sets lead us to focus on the longer term adaptations to ECC versus CON in relation to MPS. Deuterium oxide ($D_2O$) stable isotope technique was used (Robinson et al. 2011; Gasier et al. 2012) and the application has been recently validated by our group for assessment of MPS over an 8-day RET protocol (Wilkinson et al. 2014) in addition to a 6-week protocol (Brook et al. 2015). The design of the present study did not allow us to obtain any data on rest situation over time, as the volunteers trained both legs; however, the values of a similar rest situation are reported in Brook et al. (2015) ($8.1 \pm 0.1$ g*day$^{-1}$, calculated over a 6-week period).

We hypothesized that MPS would be preferentially increased in the distal VL in response to ECC, and increases in midbelly would be greater following CON. In the event, we showed that MPS was not different between ECC and CON (Fig. 5). These data are somewhat supported by a trends for a positive relationship between MPS

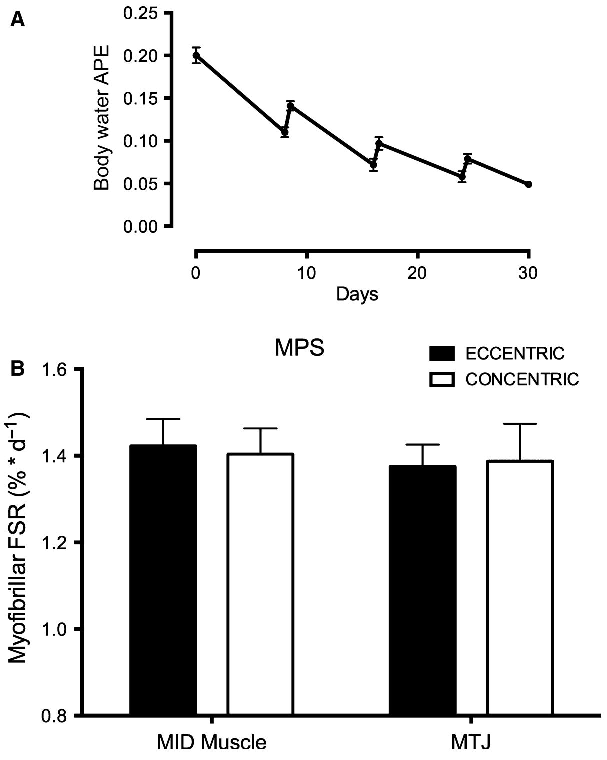

**Figure 5.** (A) Time course of body water enrichment over the 4-week training period. Values are presented as means ± SEM. (B) Myofibrillar protein synthesis rates for ECC and CON groups over the 4-week training period calculated from muscle biopsies collected at MID and MTJ sites of VL muscle. Values are presented as means ± SEM.

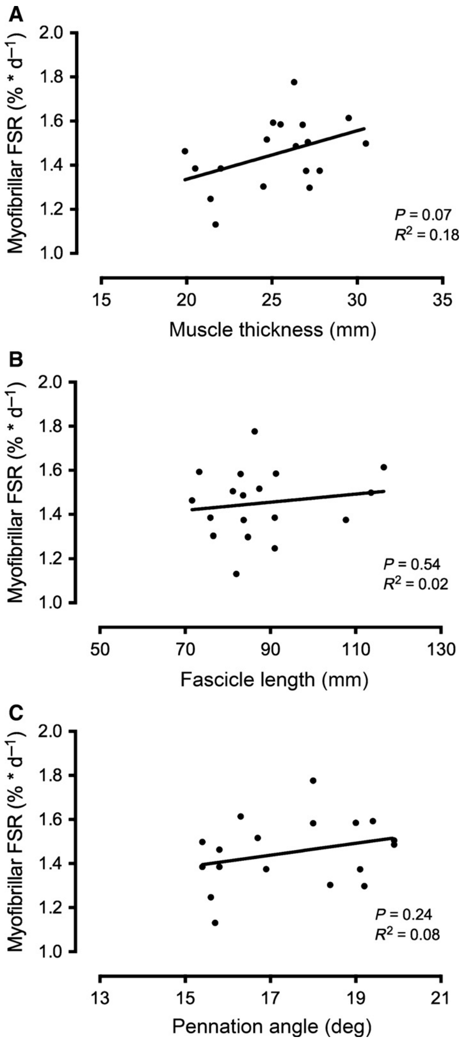

**Figure 6.** (A) Linear regression analysis between muscle thickness (at 4 weeks time point) and MID belly myofibrillar FSR (calculated between 0 and 4 weeks of either CON or ECC RET) ($R^2$ = 0.17, $P$ = 0.07). (B) Linear regression analysis between Lf and MID belly myofibrillar FSR ($R^2$ = 0.02, $P > 0.05$). (C) Linear regression analysis between PA and MID belly myofibrillar FSR ($R^2$ = 0.08, $P > 0.05$).

and MT following 4 weeks RET (Fig. 6). This is similar to a recent report of ours demonstrating a positive correlation between MT and rates of MPS (in the exercised but not rest leg) after 3 weeks of conventional unilateral RET (Brook et al. 2015). While we did not have untrained time controls in this study, this correlation would hint that MPS was elevated over this period in response to both ECC and CON protocols. We did not measure regional changes in muscle thickness in the present study (i.e., at the MTJ site). We agree this could represent a limitation of the study; however, regional differences in ACSA between the same two protocols were expected to be seen, as ECC and CON have been shown to lead to different regional hypertrophy (Seger et al. 1998). Preferential augment of ACSA, occurring closer to the end of the muscle, was prevalently brought by increases in Lf in response to ECC, whereas greater ACSA, occurring in the muscle midbelly region in response to CON, was promoted by increases in PA (Franchi et al. 2014).

However, in the present study, we were unable to distinguish MPS between MTJ and midbelly regions in response to ECC or CON. These data suggest that assembly of newly formed sarcomeres could be independent from the quantity of new contractile material synthesized. Interestingly, Fujita et al. (2007) previously demonstrated that de novo sarcomere assembly in C2C12 myotubes is possible without the requirement for newly synthesized proteins. Following electric pulse stimulation (EPS), myotubes efficiently reorganized into contractile units even in the presence of cyclohexamide (an inhibitor of protein synthesis via impairing A-P site ribosomal translocation), that is, reorganization of contractile proteins can occur independently of gross MPS processes. This suggestion is somewhat supported by the fact that myofibrillar FSR values showed trends of positive correlation only with MT

($P = 0.07$) and not with Lf and PA. Moreover, in the absence of measures of muscle protein breakdown, and without identification of plausible molecular signals regulating distinct contractile protein deposition, we can only conclude that differential adaptations localized to the muscle midbelly and distal MTJ in response to ECC and CON cannot be determined merely through quantifying longer term MPS responses.

Although it was beyond the aim of the present study, a previous study showed that the activation status of MAP kinases molecular pathways was associated with different sarcomere deposition patterns in cardiac muscle (Kehat et al. 2011). Furthermore, investigations on both animal (Martineau and Gardiner 2001; Wretman et al. 2001) and human muscle (Franchi et al. 2014) have showed that the MAPKs activation results greater after ECC contractions. Future work should focus on identifying the influence of these pathways in sarcomere assembly in skeletal muscle.

We have shown that despite the ~1.3-fold greater training load of the ECC group, gains in muscle mass and strength were similar to those observed after CON. However, similar hypertrophy is achieved through distinct architectural remodeling suggesting preferential (but not unique) addition of sarcomere in series with ECC RET and in parallel following CON training, although these distinct architectural adaptations were not reflected in regional MPS responses. Thus, contrary to prevailing views, similar hypertrophy occurs in response to ECC versus CON, and these changes are detectable in the first few weeks of a RET program; a facet reflected by similar increases in MPS (related to increased MT) following just 4 weeks of ECC and CON RET. The data might suggest that the manner of contractile protein deposition is independent to the absolute quantity of MPS in human skeletal muscle. Nonetheless, the molecular underpinnings of these divergent architectural adaptations to ECC and CON remain to be determined.

### Acknowledgments

The authors thank Margaret Baker and Amanda Gates for their technical support and assistance during the data collection and Matthew Brook for his precious assistance with data analysis of MPS.

### Conflict of Interest

None declared.
